# In‐Field Validity and Inter‐Unit Variability of Metabolic Carts During Simulated Exercise

**DOI:** 10.1111/sms.70297

**Published:** 2026-05-06

**Authors:** Bas Van Hooren, Tjeu Souren, Bart C. Bongers

**Affiliations:** ^1^ Department of Nutrition and Movement Sciences Institute of Nutrition and Translational Research in Metabolism (NUTRIM), Maastricht University Maastricht the Netherlands; ^2^ Independent Consultant Utrecht the Netherlands; ^3^ Department of Surgery Institute of Nutrition and Translational Research in Metabolism (NUTRIM), Maastricht University Maastricht the Netherlands

**Keywords:** accuracy, cardiopulmonary exercise testing, metabolic cart, precision, reliability, repeatability, simulation, validity

## Abstract

The present study had three main objectives: (a) to evaluate the in‐field validity of different commercially available cardiopulmonary exercise testing (CPET) systems when used by end‐users following typical calibration procedures, (b) to measure the variability in accuracy among identical CPET units, and (c) to explore the relationship between the age of the units, as well as the maintenance practices, and their measurement accuracy. Fifty‐seven CPET systems, calibrated and operated by end‐users in clinical practice, research, or sports settings, were assessed against a metabolic simulator that simulates breath‐by‐breath gas exchange. The values measured by each system [minute ventilation (V̇E), oxygen uptake (V̇O_2_), carbon dioxide production (V̇CO_2_), and respiratory exchange ratio (RER)] were compared to the simulated values to evaluate the accuracy. Absolute percentage errors during the simulations ranged from 1.41% to 24.6% for V̇E, 3.29%–10.6% for V̇O_2_, 2.86%–13.3% for V̇CO_2_, and 1.90%–10.0% for RER. Inter‐unit variability (%) ranged from 1.98% to 12.7% for V̇O_2_, 1.49%–8.10% for V̇CO_2_, and 1.93%–4.24% for RER. No consistent relationship between system age and accuracy was observed, nor between annual maintenance and accuracy. The validity of metabolic carts for measuring respiratory gas variables varied significantly even between identical systems, despite passing manufacturers' calibration checks. Furthermore, inter‐unit variability of most systems exceeded intra‐unit test‐retest variability, thus necessitating caution when using devices interchangeably, as this may increase measurement noise, even within the same laboratory. Most inaccuracies seemed related to technological errors, although some user errors were also identified, indicating the need for a holistic approach to identify errors.

## 1 Introduction

Cardiopulmonary exercise testing (CPET) measures variables such as oxygen uptake (V̇O_2_), carbon dioxide production (V̇CO_2_), and minute ventilation (V̇E) to assess cardiovascular, pulmonary, and metabolic function during progressive exercise up to volitional exhaustion. Accurate measurement of respiratory gas indices is critical because they are used to inform clinical decisions [1, 2, 3, 4], for exercise training prescriptions [5, 6], and to support a wide range of research applications [7, 8, 9, 10, 11, 12, 13, 14, 15, 16].

Metabolic simulation over recent years has become the gold standard for evaluating the accuracy and reliability of CPET systems, as it provides precise control of gas flows and concentrations under highly standardized conditions that mimic the metabolic exercise response, thereby eliminating biological variability [17, 18, 19, 20, 21, 22, 23, 24, 25, 26]. Using this method, the accuracy and reliability of numerous CPET systems have been documented [17, 18, 20, 24, 27, 28, 29, 30, 31, 32]. Most CPET validation studies examine a single system/unit (defined here as an individual physical measurement device belonging to the same model [i.e., a specific product design and hardware‐software configuration marketed under a single model name by a manufacturer]) of a specific brand and model, implicitly assuming: (a) the results apply to all units of that model, and (b) the operating procedures used during well‐controlled studies, along with the resulting accuracy, are also representative of in‐field (e.g., clinical exercise laboratory) operating procedures and accuracy. Both assumptions, however, can be challenged.

First, although studies evaluating technological variability between identical units (i.e., inter‐unit variability) report this variability to be relatively low (CV's typically < 1.5%) [19, 21, 24, 33], all studies examining inter‐unit differences used only two units of each model. Therefore, it remains unclear whether the observed accuracy and variability represent those in a larger sample of units. Additionally, in at least two studies [19, 21], the units assessed were new and very recently obtained from the manufacturer. Over time, components such as oxygen and carbon dioxide analyzers may degrade, leading to increased inter‐unit differences. Although most manufacturers offer regular service and maintenance intervals (involving for example hardware inspection and cleaning of gas sampling lines, water traps, Nafion tubing), the impact of system aging and maintenance practices on measurement accuracy has received limited empirical attention.

Second, in all previous studies assessing inter‐unit technological variability [19, 21, 24, 33], the units were calibrated and operated by experienced technical research staff. While such conditions optimize unit accuracy and reliability, they do not necessarily reflect in‐field clinical or sports science practices where users may follow different, and potentially less rigorous, calibration and operating procedures. These operational inconsistencies may influence measurement accuracy, yet the real‐world impact remains poorly quantified. In partial support, studies have documented possibly erroneous CPET data, despite following manufacturer calibration guidelines and adhering to periodic quality control [34, 35, 36]. Xu et al. [34] for instance performed a daily metabolic simulation assessment on eight different CPET systems from six manufacturers over the course of 5 years. Despite meeting the volume and gas concentration calibration checks as indicated by the manufacturer's software, the systems still failed between 20% and 90% of the metabolic simulator validation assessments. This may be because separate gas‐ and volume‐calibration procedures do not fully capture the combined accuracy of V̇E, V̇O_2_, and V̇CO_2_.

Overall, these observations suggest that findings obtained under ideal laboratory conditions on a single unit cannot necessarily be generalized to other units and in‐field performance. Furthermore, they emphasize the need for a large‐scale study to evaluate inter‐unit variability across a range of research labs and clinical exercise laboratories. Given these gaps, the present study had three aims: (a) to assess the in‐field validity of multiple commercially available CPET systems when operated by end‐users following typical calibration procedures, (b) to evaluate inter‐unit variability in the accuracy of multiple identical CPET models, and (c) to examine the relationship between unit age and maintenance practices and measurement accuracy. Understanding inter‐unit differences (whether due to technological or operational factors) is particularly relevant in the context of multicenter studies, where data collected across multiple sites using the same CPET model are pooled and compared. Large‐scale initiatives such as the FRIEND registry [37, 38] and the HERITAGE Family Study [39, 40] highlight the increasing prevalence of such designs [35, 36, 41]. Similarly, it is crucial for clinical centers or laboratories using multiple units of the same model and for comparing physiological data across studies conducted in different labs. In support of the latter, a recent attempt to establish an all‐age reference equation by combining data across different CPET studies, showed that CPET outcomes significantly differed between CPET systems, and measurement site [42]. Finally, knowledge of the in‐field accuracy of different CPET systems is essential for a better understanding of the need for more rigorous quality control procedures.

## 2 Methods

### 2.1 General Study Design

This study was a cross‐sectional observational study in which different CPET systems were assessed for accuracy by comparing their measured values against those simulated with a metabolic simulator. For this purpose, one researcher (TS) visited multiple hospitals/rehabilitation centers, university research labs, sports physicians, and other CPET users in the Netherlands, Belgium, and Germany, and assessed the accuracy of each CPET system during simulated exercise with a metabolic simulator. In some situations where a system failed to meet the simulation assessment criteria for acceptable results (see later), the CPET system was reassessed to verify its performance and accuracy after repair/maintenance. Some example cases of such reassessments are discussed with the intention of raising awareness among CPET users about common causes of malfunctioning equipment.

### 2.2 Equipment

CPET data were collected from a total of 57 different CPET systems from six different manufacturers across 30 different facilities (Table 1). For all but two systems (MasterScreen CPX and MetaLyzer 3B), a unit from a model similar to the model included in the present study has also been investigated in previous studies under controlled laboratory conditions [18, 22]. The manufacturers of the CPET systems or the metabolic simulator had no role in the study design, analysis, or interpretation of the data collected, nor in the writing of the report or the decision to submit the paper for publication.

**TABLE 1:** Overview of the CPET systems measured in this study.

Manufacturer	Number of units assessed per model	User setting per model
Jaeger Medical, Mettawa, IL, USA*	Vyntus CPX (*n* = 11; all B × B mode) MasterScreen CPX (*n* = 1)	Sports physician (*n* = 1), Hospital/rehabilitation center (*n* = 10) Hospital/rehabilitation center (*n* = 1)
COSMED, Rome, Italy	Quark CPET (*n* = 8; all B × B mode) K5 (*n* = 1; B × B mode)	Sports physician (*n* = 5), Hospital/rehabilitation center (*n* = 2), University research laboratory (*n* = 1) University research laboratory (*n* = 1)
Cortex Biophysik, Leipzig, Germany	MetaLyzer 3B (*n* = 15; all B × B mode) MetaMax 3B (*n* = 5; all B × B mode)	Sports physician (*n* = 3), Hospital/rehabilitation center (*n* = 3), University research laboratory (*n* = 8), Distributor (*n* = 1) Hospital/rehabilitation center (*n* = 3), University research laboratory (*n* = 1), Distributor (*n* = 1)
Geratherm Respiratory GmbH, Bad Kissingen, Germany	Ergostik (*n* = 10; all B × B mode)	Hospital/rehabilitation center (*n* = 10)
VO2 Master Health Sensors Inc., Vernon, BC, Canada	VO2Master (*n* = 3; all B × B mode)	University research laboratory (*n* = 3)
ENDO Medical, Palo Alto, CA, USA	PNOĒ (*n* = 3; all B × B mode)	Sports trainer (*n* = 3)

Abbreviations: B × B = breath‐by‐breath, CPET = cardiopulmonary exercise testing.

* Previously Vyaire Medical.

### 2.3 Metabolic Simulator

The human gas‐exchange response during exercise was mimicked using a state‐of‐the‐art metabolic simulator comprising a breathing simulator and a gas‐infusion system (Relitech Systems BV, Nijkerk, The Netherlands). This system is reliable and produces highly precise breath‐by‐breath variables [17, 18]. A detailed description of the simulator can be found elsewhere [17, 18]; therefore, a concise description will be provided here. Briefly, the breathing simulator uses a motorized syringe (piston) to simulate breathing variables and can also simulate different gas concentrations by pumping room air back and forth and injecting amounts of pure CO_2_ and N_2_ (purity ≥ 99.99%; Linde Gas, Netherlands). The injection of known quantities of pure CO_2_ generates exhaled breaths that simulate a precise volume of V̇CO_2_ at different breathing frequencies. Similarly, by injecting pure N_2_, the ambient air O_2_ content is reduced to a specific O_2_ concentration to simulate V̇O_2_ rates. The simulated V̇O_2_ and V̇CO_2_ are automatically calculated as detailed previously [18].

The ratio between V̇CO_2_ and V̇O_2_ (i.e., RER) can also be set to vary between 0.75 and 1.05. The amount of injected CO_2_ and N_2_ during each breath exhaled by the metabolic simulator is regulated by high‐precision mass flow controllers, resulting in a precision of < 0.2% for the simulated O_2_ and CO_2_ fractions. Combined with the simulator's volume‐stroke accuracy, the metabolic simulator produces V̇O_2_ and V̇CO_2_ with < 0.5% error, even at high ventilation levels. Further, when environmental conditions are identical, the between‐session variability in simulated V̇O_2_ and V̇CO_2_ is < 0.025% and < 0.005%, respectively [22]. The simulator was certified by the manufacturer before the first in‐field test day and again after the last system was tested for this study.

### 2.4 Simulation Protocol

The CPET systems were connected directly to the metabolic stimulator outlet, using custom‐made adaptors when required. It was attempted to use the same dead space for all systems, and to minimize turbulation introduced by custom‐made adaptors. Each CPET system underwent a standardized protocol to assess V̇E, V̇O_2_, V̇CO_2_, and RER as primary outcomes.

The “Std” mode on the simulator was used first, with the tidal volume set at 2 L and RER at 1.00 (V̇O_2_, V̇CO_2_ equal). In this mode, breathing frequencies (BF) changes from 20 to 40, 60, and 80·min^−1^. V̇O_2_ and V̇CO_2_ at each breathing frequency were 1, 2, 3, and 4 L·min^−1^. BF and tidal volume were used to mimic physiological values reported during human physical activity and exercise testing [43, 44, 45, 46, 47]. A second protocol was performed in “CPX” mode to simulate increasing RERs with rising BFs and ventilation, as in common human CPET. The RER variations were performed to mimic the increased oxidation of carbohydrates with increasing exercise intensity and to mimic the buffering of [H^+^] by bicarbonate ([HCO_3_^−^]) at high exercise intensities [48]. The simulated RER values were 0.75, 0.85, 0.95, and 1.05, with V̇O_2_ being 1, 2, 3, and 4 L·min^−1^ at each RER, with a corresponding V̇CO_2_ of 0.75, 1.7, 2.85, and 4.2 L·min^−1^. Note that the lowest step of the CPX procedure (i.e., with BF of 10·min^−1^, RER 0.75, and V̇O_2_ of 1 L·min^−1^) required a separate mask adapter for VO2Master Pro, and this stage was therefore simulated separately while using this mask. Each stage lasted at least 2 min to ensure sufficient time for breath collection stabilization. The graphical user interface for each system was checked to confirm steady‐state recordings for each stage.

### 2.5 Data Collection Settings for Each CPET System

The metabolic simulator mimics human breathing and produces artificial breaths that are highly accurate and repeatable. The mass‐flow controllers used in the metabolic simulator for CO_2_ and N_2_ have a temperature‐controlled output normalized to absolute volume output in standard temperature and pressure dry (STPD) (SLN, normalized standard liters), as detailed previously [18]. Ventilation, the volume strokes from the piston pump of the metabolic simulator, uses room air, and is thus at ambient conditions (ambient temperature and pressure; ATP).

CPET systems are typically used for human testing. Human expired volumes have a higher temperature and humidity than ambient air; and it is common to express the expired lung volumes in saturated body temperature and pressure conditions (BTPS). By measuring or assuming a specific humidity, temperature, and pressure of the expired air, the CPET systems convert measured breath values to BTPS and V̇O_2_, V̇CO_2_ to STPD units to allow comparison between different measurement conditions. Most CPET systems typically assume the expired gas is 100% humidified and has a temperature of 31.5°C. Since this assumption is incorrect during the metabolic simulation experiments, the gas volumes in STPD must be corrected to allow comparison with the simulated values. Therefore the “BTPS correction” was turned off within the software application when possible (for manufacturers enabling this [Jaeger Medical, VO2Master Pro]), or we converted the measured values to allow comparison.

Room temperature and relative humidity ranged between 19°C and 24°C and 40%–70%, respectively, during all simulation measurements. During all experiments, the room was ventilated as it would be during normal local day‐to‐day operational usage.

### 2.6 CPET Calibration

Each CPET system was calibrated twice by the local staff using the calibration protocol they would generally use before a CPET test. A double calibration procedure was performed to reduce the possibility of sensor drift due to inadequate stabilization. The calibration typically involved a warm‐up period of approximately 1 h before performing a gas calibration using certified calibration gases and a volume calibration using a certified 3 L syringe (or using the automated method within the system [i.e., Vyntus CPX]). The only exception to this is PNOĒ, which states that only room air calibration is required for routine purposes. The use of certified calibration gas is optional and not a standard practice for PNOĒ devices. However, in previous work, we found a substantial improvement in the accuracy (albeit still relatively poor performance) when calibration gases were used [18]. Therefore, in the tests for this study, the PNOĒ system was only assessed after calibration with certified gas, using a CO_2_/O_2_ mix (5% CO_2_/16% O_2_) to calibrate the CO_2_/O_2_ sensor. All systems passed the manufacturer‐calibration checks before the initiation of the simulation protocol.

### 2.7 Data Processing

During the simulation tests, the mean value from the last minute of each stage was used for analysis to ensure adequate flushing of the gas‐filled dead space in the simulator. The period selected for analysis was also confirmed by visual inspection of a steady state.

### 2.8 Statistical Analysis

The accuracy of the CPET systems was assessed for the main ventilatory and gas exchange variables: V̇E (L·min^−1^), V̇O_2_ (mL·min^−1^), V̇CO_2_ (mL·min^−1^), and RER.

The agreement between the CPET systems and the metabolic simulator was assessed in several ways. First, the measurement error in original units was calculated for the simulation test by subtracting the expected value (i.e., the simulated value) from the measured value (i.e., the converted CPET readouts). This error was also expressed as a percentage of the expected value (i.e., [(measured–expected)/expected] × 100) and used to compute the mean relative percentage error and mean absolute percentage error for all simulation steps of each system to indicate the overall measurement error. Error thresholds for the main ventilatory and gas exchange variables were defined as follows: good, when < 3%; acceptable, when < 5%; and poor, when ≥ 5%. This classification aligns with previous research and the 3% error specified by most manufacturers for these outcomes [18], and is also approximately consistent with the < 3% error being considered acceptable for volume measurements according to the 2019 American Thoracic Society and European Respiratory Societies guidelines [49]. To investigate whether the typical user setting influences CPET system accuracy, we assessed the accuracy across all systems for a specific user setting: (a) hospital/rehabilitation centers, (b) university research labs, (c) sports physicians, and (d) sports trainers. Two systems in the present study were used for demonstration/training purposes and located at a distributor, and these were not included as a separate category because this does not represent a typical user setting.

Inter‐unit variability was quantified by first computing the standard deviation of the values measured across units for each simulated step. This standard deviation was subsequently divided by the mean of the measured values and multiplied by 100 to express it as a percentage (i.e., coefficient of variation; CV). The CVs were then averaged across all steps and both simulation protocols to indicate the overall CV.

For brands with at least two units of a specific model available, we also investigated whether the relative percentage error in the primary ventilatory and gas‐exchange outcomes changed at higher simulated values. To this purpose, the relative percentage errors were averaged for each respective simulation step of the Std and CPX protocols. A linear mixed model with a fixed effect for each step and a random slope and intercept per unit was subsequently applied to assess if the slope of the regression line fitted to the error differed significantly from zero.

Finally, a linear regression analysis was used to investigate the relationship between system age and the overall absolute percentage error across all simulated steps within each system. Influential outliers were identified using a combination of Cook's distance and visual inspection of scatterplots. A linear mixed model was used to investigate the overall relationship across all systems, with system age as a fixed factor and a random intercept and slope per system. An independent *t*‐test was used to assess if the overall absolute percentage error differed between systems receiving yearly maintenance and those that did not. Normality of the residuals was inspected visually for all analyses.

## 3 Results

Overall, 57 unique CPET units were assessed across 30 different locations. Thirteen systems were assessed at least twice, but only the first assessment was included in the primary analyses. Users per unique system included sports physicians (*n* = 9), hospitals/rehabilitation centers (*n* = 29), university research labs (*n* = 14), sports coaches (*n* = 3), and distributors (*n* = 2). Mean ± SD system age at the time of assessment was 3.97 ± 2.61 years. Of the systems assessed, 77.6% received standard yearly maintenance, 6.9% received maintenance on request, and 15.5% received no yearly maintenance.

**FIGURE 1:**
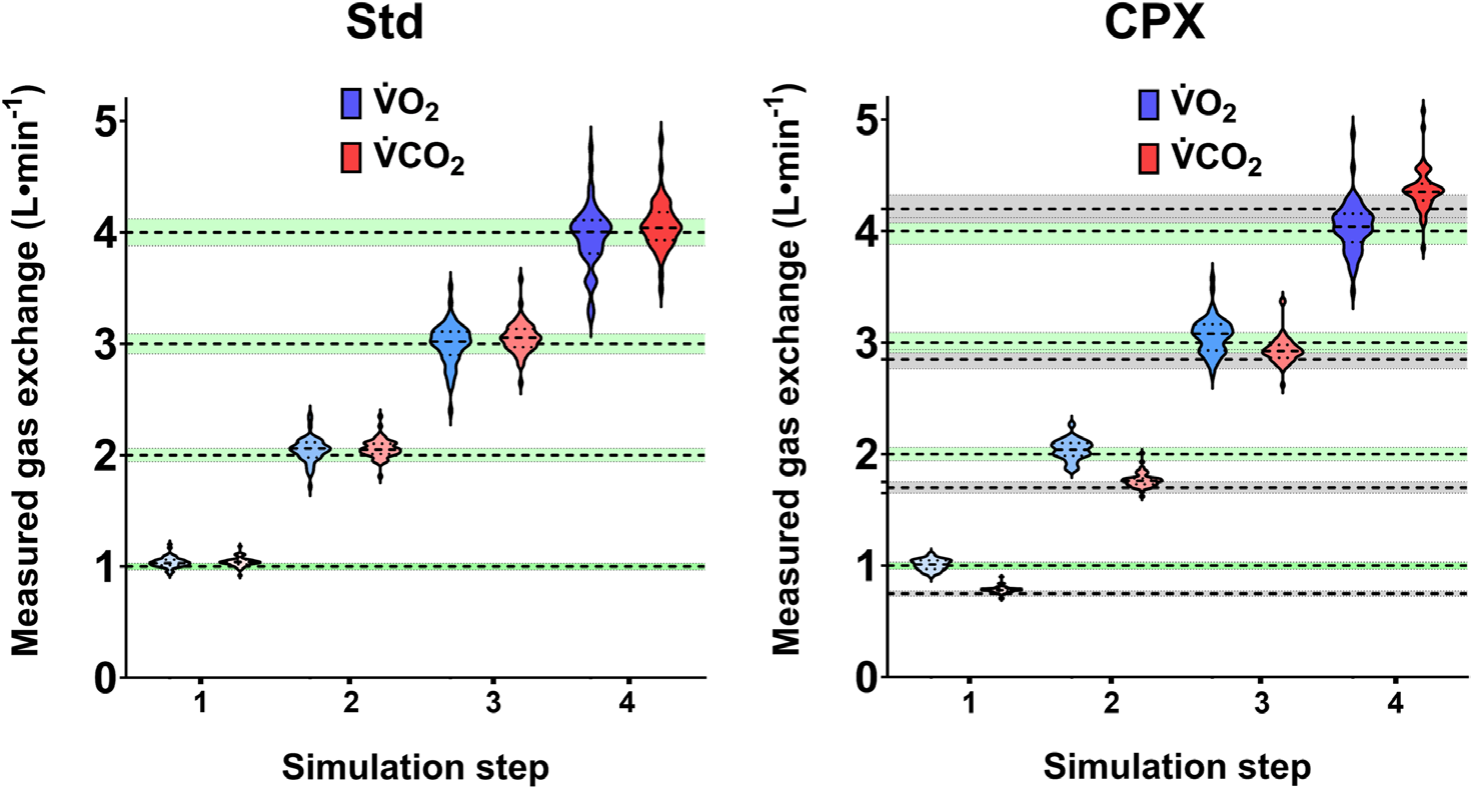
Violin plots show the mean gas exchange values obtained from each individual CPET unit, pooled across all units at each simulation step in the “Std” (left: RER = 1.00) and “CPX” (right: RER increases from 0.75 to 0.85, 0.95 and finally 1.05) modes of the simulator. Shaded areas depict the ±3% range considered good agreement between the CPET system and the simulated values. In the Std mode, the expected value and error range are similar for V̇O_2_ and V̇CO_2_; therefore, only one error band is shown. On the right side, the green shaded area represents the ±3% area for V̇O_2_, and the gray area represents the ±3% band for V̇CO_2_. Dashed lines and dotted lines within each violin represent the median and interquartile range, respectively. RER = respiratory exchange ratio, V̇CO_2_ = carbon dioxide production, V̇O_2_ = oxygen uptake.

**FIGURE 2:**
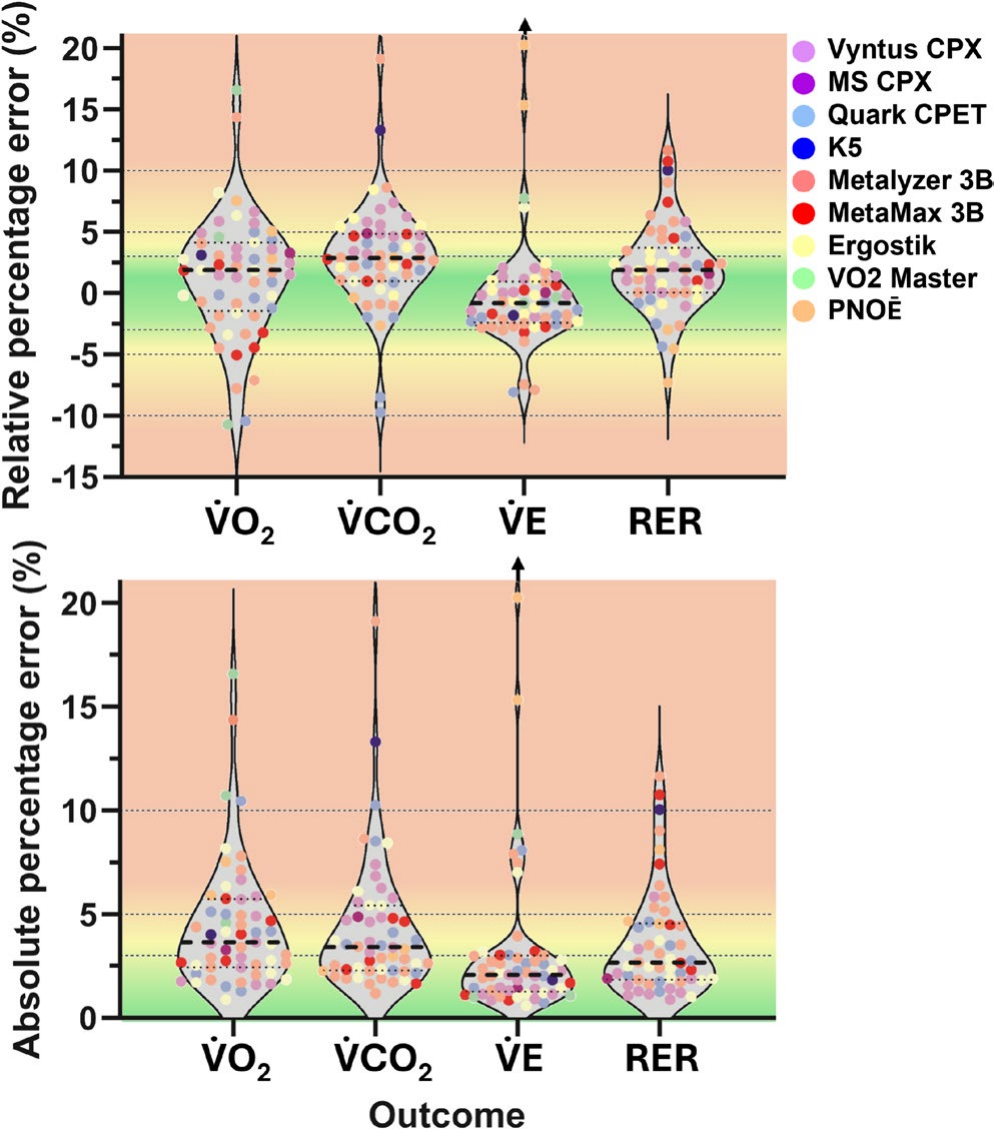
Relative (top) and absolute (bottom) percentage errors per outcome averaged over all steps within each simulation mode (“Std” and “CPX”) and subsequently averaged over both modes. Each dot depicts the mean percentage error for one system. Each brand and model is color coded. Note that there is one system (PNOĒ), which had a relative and absolute percentage error of 38% for V̇E, which was beyond the scale of the axis chosen to maintain readability for the other datapoints. Dashed lines within each violin represent the median and interquartile range. Dashed lines in the background present the thresholds for good (within < 3%), acceptable (within < 5%) agreement, and an additional threshold at 10% indicating very poor accuracy. RER = respiratory exchange ratio, V̇CO_2_ = carbon dioxide production, V̇E = minute ventilation, V̇O_2_ = oxygen uptake.

**FIGURE 3:**
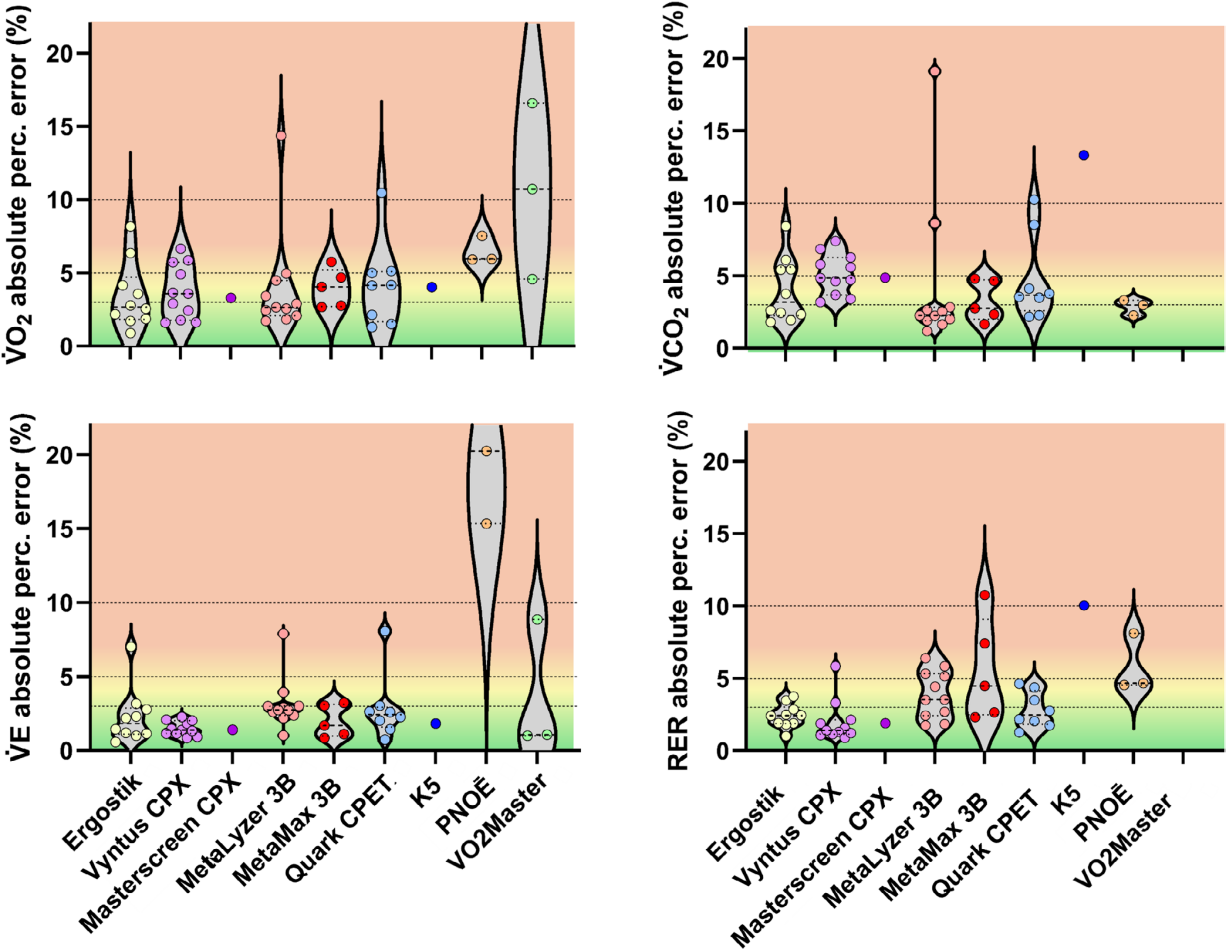
Mean absolute percentage errors per system. Each dot represents the mean error across all simulated steps for one unique system. Dashed lines in the background indicate thresholds for good (within < 3%) and acceptable (within < 5%) agreement, with an additional threshold at 10% indicating very poor accuracy. RER = respiratory exchange ratio, V̇CO_2_ = carbon dioxide production, V̇E = minute ventilation, V̇O_2_ = oxygen uptake.

The mean gas‐exchange values recorded by each system at each step of the Std and CPX protocols are shown in Figure 1. Relative and absolute percentage errors averaged over all simulated volumes are listed in Table 2 and illustrated in Figure 2. Table 3 presents the number of systems with an overall mean absolute percentage error below pre‐defined cut‐offs. Inter‐unit variability is shown in Table 4. Figure 3 displays mean percentage errors for each system.

**TABLE 2:** Mean ± SD and range of relative and absolute percentage errors for the primary respiratory gas exchange outcomes.

	V̇O_2_ (min–max) [%e]	V̇CO_2_ (min–max) [%e]	V̇E (min–max) [%e]	RER (min–max) [%e]
**Relative errors**				
Overall (*n* = 57)^b^	1.39 ± 4.95 (−19.9 to 21.8)	2.96 ± 4.36 (78.0 to 20.9)	0.36 ± 6.75 (−11.0 to 41.1)	2.00 ± 3.7 (−11.0 to 24.7)
Hospitals/rehabilitation centers (*n* = 29)	1.72 ± 4.67 (−11.8 to 10.3)	3.67 ± 4.50 (−5.28 to 11.7)	−0.01 ± 5.47 (−7.85 to 10.9)	2.02 ± 3.8 (−7.10 to 18.0)
Research labs (*n* = 14)	0.59 ± 7.93 (−19.9 to 21.8)	4.16 ± 7.37 (−12.7 to 20.9)	−1.78 ± 3.30 (−9.40 to 11.6)	4.61 ± 3.55 (−2.40 to 24.7)
Sports physicians (*n* = 9)	0.82 ± 3.86 (−11.1 to 10.2)	0.85 ± 4.71 (−78.0 to 8.48)	−2.50 ± 3.16 (−11.0 to 5.80)	1.30 ± 3.75 (−8.00 to 12.3)
Sports trainers (*n* = 3)	5.13 ± 2.37 (−9.58 to 13.1)	−0.04 ± 2.79 (−7.07 to 7.90)	24.6 ± 11.9 (8.85 to 41.1)	−4.95 ± 2.19 (−11.0 to 6.67)
Vyntus CPX (*n* = 11)	3.63 ± 1.92 (−1.38 to 8.40)	5.12 ± 1.39 (2.00 to 9.10)	0.39 ± 1.33 (−3.58 to 9.20)	1.49 ± 1.84 (−3.16 to 8.70)
MasterScreen CPX (*n* = 1)	3.29 ± n.a. (0.83 to 5.33)	4.87 ± n.a. (1.47 to 8.29)	0.04 ± n.a. (−2.50 to 5.50)	1.57 ± n.a. (−1.33 to 4.00)
Quark CPET (*n* = 8)	0.10 ± 4.83 (−18.8 to 9.20)	−1.27 ± 5.38 (78.0 to 8.13)	−2.44 ± 2.38 (−11.0 to 3.50)	0.01 ± 2.84 (−8.00 to 12.0)
K5 (*n* = 1)	3.09 ± n.a. (−2.73 to 9.53)	13.3 ± n.a. (8.00 to 17.8)	−1.82 ± n.a. (−3.55 to −0.76)	10.0 ± n.a. (6.40 to 17.7)
MetaLyzer 3B (*n* = 15)	−0.30 ± 5.33 (−11.8 to 17.0)	3.03 ± 5.03 (−6.38 to 20.9)_	−2.65 ± 2.52 (−9.48 to 5.80)	3.59 ± 3.78 (−8.00 to 17.3)
MetaMax 3B (*n* = 5)	−1.72 ± 3.53 (−17.7 to 7.40)	3.11 ± 1.62 (−1.88 to 7.86)	−1.35 ± 1.73 (−4.73 to 3.95)	5.19 ± 3.95 (−4.00 to 24.7)
Ergostik (*n* = 10)	1.97 ± 3.57 (−7.10 to 10.3)	3.45 ± 2.84 (−5.28 to 11.7)	0.37 ± 2.86 (−7.85 to 10.9)	1.48 ± 1.75 (−7.10 to 9.47)
VO2Master (*n* = 3)	3.48 ± 13.7 (−19.9 to 21.8)	—	2.19 ± 4.81 (−4.45 to 11.6)	—
PNOĒ^a^ (*n* = 3)	5.13 ± 2.37 (−9.58 to 13.1)	−0.04 ± 2.79 (−7.07 to 7.90)	24.6 ± 12.0 (8.85 to 41.1)	−4.95 ± 2.19 (−11.0 to 6.67)
**Absolute errors**				
Overall (*n* = 57)^b^	4.44 ± 3.05 (0.00 to 21.8)	4.34 ± 3.18 (0.00 to 78.0)	3.63 ± 5.77 (0.00 to 41.1)	3.58 ± 2.56 (0.00 to 24.7)
Hospitals/rehabilitation centers (*n* = 29)	3.49 ± 2.86 (0.00 to 11.8)	4.08 ± 3.77 (0.00 to 11.7)	1.95 ± 4.37 (0.00 to 10.9)	2.87 ± 2.75 (0.00 to 18.0)
Research labs (*n* = 14)	6.35 ± 4.73 (0.20 to 21.8)	6.08 ± 5.78 (0.00 to 20.9)	3.09 ± 2.40 (0.1 to 11.6)	4.99 ± 3.09 (0.10 to 24.7)
Sports physicians (*n* = 9)	4.14 ± 1.81 (0.00 to 11.1)	4.14 ± 2.67 (0.00 to 78.0)	3.05 ± 2.75 (0.00 to 11.0)	3.32 ± 2.46 (0.00 to 12.3)
Sports trainers (*n* = 3)	6.46 ± 0.93 (1.40 to 31.1)	2.86 ± 0.53 (0.18 to 7.90)	24.6 ± 11.9 (8.9 to 41.1)	5.77 ± 2.03 (2.00 to 11.0)
Vyntus CPX (*n* = 11)	3.70 ± 1.83 (0.00 to 8.40)	5.12 ± 1.39 (2.00 to 9.10)	1.49 ± 0.49 (0.00 to 9.20)	1.98 ± 1.45 (0.00 to 8.70)
MasterScreen CPX (*n* = 1)	3.29 ± n.a. (0.83 to 5.33)	4.87 ± n.a. (1.47 to 8.29)	1.41 ± n.a. (0.05 to 5.50)	1.90 ± n.a. (0.00 to 4.00)
Quark CPET (*n* = 8)	4.22 ± 2.94 (0.03 to 18.8)	4.77 ± 2.97 (0.00 to 78.0)	2.85 ± 2.23 (0.12 to 11.0)	2.81 ± 1.23 (0.00 to 12.0)
K5 (*n* = 1)	4.02 ± n.a. (1.00 to 9.53)	13.3 ± n.a. (8.00 to 17.8)	1.82 ± n.a. (0.76 to 3.55)	10.0 ± n.a. (6.40 to 17.7)
MetaLyzer 3B (*n* = 15)	4.38 ± 3.31 (0.15 to 17.0)	3.80 ± 4.58 (0.00 to 20.9)	3.14 ± 1.97 (0.02 to 9.48)	4.55 ± 2.83 (0.00 to 17.3)
MetaMax 3B (*n* = 5)	3.98 ± 1.31 (0.08 to 17.7)	3.24 ± 1.41 (0.00 to 7.86)	1.98 ± 1.09 (0.05 to 4.73)	5.53 ± 3.55 (0.00 to 24.7)
Ergostik (*n* = 10)	3.42 ± 2.27 (0.10 to 10.3)	4.02 ± 2.22 (0.10 to 11.7)	2.28 ± 1.86 (0.00 to 10.9)	2.41 ± 0.81 (0.00 to 9.47)
VO2Master (*n* = 3)	10.6 ± 6.00 (0.53 to 21.8)	—	3.64 ± 4.51 (0.21 to 11.6)	—
PNOĒ^a^ (*n* = 3)	6.50 ± 0.93 (1.40 to 13.1)	2.86 ± 0.53 (0.18 to 7.90)	24.6 ± 12.0 (8.85 to 41.1)	5.77 ± 2.03 (2.00 to 11.0)

*Note:* Some SDs are not applicable because only one unit per model was investigated in this study.

Abbreviations: n.a. = not applicable, RER = respiratory exchange ratio, V̇CO_2_ = carbon dioxide production, V̇E = minute ventilation; V̇O_2_ = oxygen uptake.

^a^ This system did not function at the highest breathing frequency in the Std step, so this step is excluded from these analyses.

^b^ Note that 2 systems at distributors were not included as a separate category.

**TABLE 3:** Number and proportion of systems having an absolute percentage error below three pre‐defined cut‐off values.

	V̇O_2_			V̇CO_2_			V̇E			RER		
	< 3%	< 5%	< 10%	< 3%	< 5%	< 10%	< 3%	< 5%	< 10%	< 3%	< 5%	< 10%
**Number**												
Overall (*n* = 57)^b^	23	41	53	24	40	51	43	49	54	30	43	51
Hospitals/rehabilitation centers (*n* = 29)	16	24	29	11	21	29	25	28	29	21	25	28
Research labs (*n* = 14)	4	9	10	6	7	9	10	12	14	4	6	9
Sports physicians (*n* = 9)	2	6	9	3	7	8	7	7	9	5	8	9
Sports trainers (*n* = 3)	0	0	3	2	3	3	0	0	0	0	2	3
Vyntus CPX (*n* = 11)	5	8	11	0	6	11	11	11	11	9	10	11
MasterScreen CPX (*n* = 1)	0	1	1	0	1	1	1	1	1	1	1	1
Quark CPET (*n* = 8)	3	6	7	2	6	7	6	7	8	5	8	8
K5 (*n* = 1)	0	1	1	0	0	0	1	1	1	0	0	0
MetaLyzer 3B (*n* = 15)	7	12	14	12	13	14	11	13	15	5	9	14
MetaMax 3B (*n* = 5)	2	4	5	3	5	5	3	5	5	2	3	4
Ergostik (*n* = 10)	6	8	10	5	6	10	8	9	10	8	10	10
VO2Master (*n* = 3)	0	1	1	0	0	0	2	2	3	0	0	0
PNOĒ (*n* = 3)	0	0	3	2	3	3	0	0	0	0	2	3
**Percentage** ^a^												
Overall (*n* = 57)^b^	40	72	93	42	70	89	75	86	95	53	75	89
Hospitals/rehabilitation centers (*n* = 29)	55	83	100	38	72	100	86	97	100	72	86	97
Research labs (*n* = 14)	29	64	71	43	50	64	71	86	100	29	43	64
Sports physicians (*n* = 9)	22	67	100	33	78	89	78	78	100	56	89	100
Sports trainers (*n* = 3)	0	0	100	67	100	100	0	0	0	0	67	100
Vyntus CPX (*n* = 11)	45	73	100	0	55	100	100	100	100	82	91	100
MasterScreen (*n* = 1)	0	100	100	0	100	100	100	100	100	100	100	100
Quark CPET (*n* = 8)	38	75	88	25	75	88	75	88	100	63	100	100
K5 (*n* = 1)	0	100	100	0	0	0	100	100	100	0	0	0
MetaLyzer 3B (*n* = 15)	47	80	93	80	87	93	73	87	100	33	60	93
MetaMax 3B (*n* = 5)	40	80	100	60	100	100	60	100	100	40	60	80
Ergostik (*n* = 10)	60	80	100	50	60	100	80	90	100	80	100	100
VO2Master (*n* = 3)	0	33	33	0	0	0	67	67	100	0	0	0
PNOĒ (*n* = 3)	0	0	100	67	100	100	0	0	0	0	67	100

Abbreviations: RER = respiratory exchange ratio, V̇CO_2_ = carbon dioxide production, V̇E = minute ventilation, V̇O_2_ = oxygen uptake.

^a^ Percentages are expressed relative to the number of systems from the specific model.

^b^ Note that 2 systems at distributors were not included as a separate category.

**TABLE 4:** Inter‐unit variability in absolute percentage errors for the main respiratory gas exchange outcomes.

	V̇O_2_ (%)	V̇CO_2_ (%)	V̇E (%)	RER (%)
**Coefficient of variation**				
Vyntus CPX (*n* = 11)	1.98	1.49	1.43	1.93
MasterScreen CPX (*n* = 1)	—	—	—	—
Quark CPET (*n* = 8)	4.93	8.10	2.62	2.86
K5 (*n* = 1)	—	—	—	—
MetaLyzer 3B (*n* = 15)	5.66	5.02	2.68	3.99
MetaMax 3B (*n* = 5)	4.06	2.01	1.91	3.90
Ergostik (*n* = 10)	3.76	3.12	3.09	2.03
VO2Master (*n* = 3)	12.7	—	4.83	—
PNOĒ (*n* = 3)	4.86	3.35	8.65	4.24

Abbreviations: RER = respiratory exchange ratio, V̇CO_2_ = carbon dioxide production, V̇E = minute ventilation, V̇O_2_ = oxygen uptake.

### 3.1 Change in Relative Error With Higher Simulated Values

**FIGURE 4:**
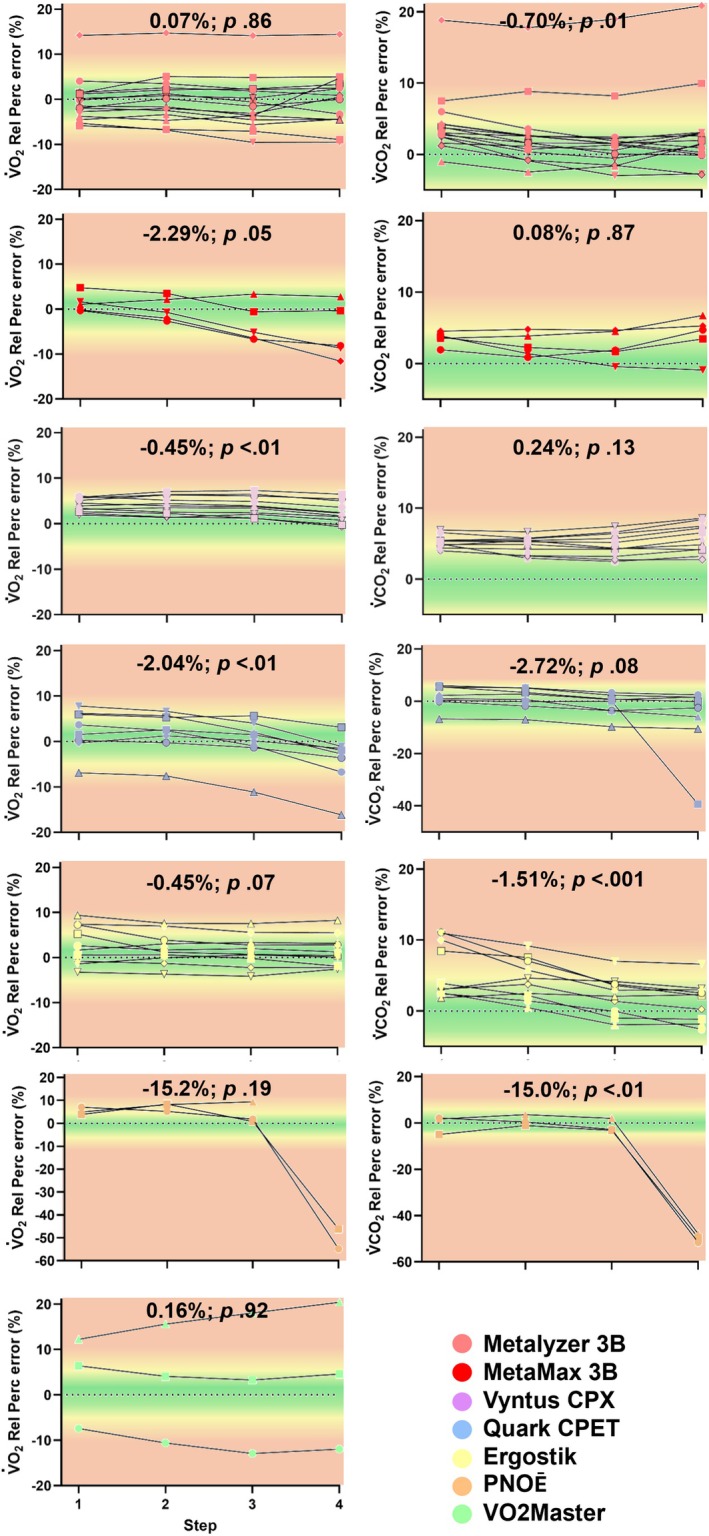
Change in relative percentage error for V̇O_2_ and V̇CO_2_ with higher simulated values. Dots depict the mean error of the first, second, third, and fourth Std and CPX step, respectively, per system. Positive values represent an overestimation relative to the simulated value, while negative values indicate an underestimation. V̇CO_2_ = carbon dioxide production, V̇O_2_ = oxygen uptake.

Table 5 presents the results of the linear mixed model evaluating whether the relative percentage error for the primary ventilatory and gas‐exchange outcomes varies with higher simulated values. Figure 4 illustrates the change in the relative percentage error across all systems.

**TABLE 5:** Outcome of the linear mixed model, which assesses whether the relative percentage error for gas exchange outcomes changes with alterations in simulated volumes.

System	Direction slope; magnitude; *p*‐value for slope of V̇E error	Direction slope; magnitude; *p*‐value for slope of V̇O_2_ error	Direction slope; magnitude; *p*‐value for slope of V̇CO_2_ error	Direction slope; magnitude; *p*‐value for slope of RER error
Vyntus CPX (*n* = 11)	↓; −0.33%; 0.04	↓; −0.45%; < 0.01	↔; 0.24%; 0.13	↑; 0.69%; < 0.001*
MasterScreen CPX (*n* = 1)	—	—	—	—
Quark CPET (*n* = 8)	↔; −0.25%; 0.31	↓; −2.04%; < 0.01*	↔; −2.72%; 0.08	↔; 0.92%; 0.07
K5 (*n* = 1)	—	—	—	—
MetaLyzer 3B (*n* = 15)	↔; 0.08%; 0.45	↔; 0.07%; 0.86	↓; −0.70%; 0.01*	↓; −0.73%; 0.05*
MetaMax 3B (*n* = 5)	↔; 0.11%; 0.70	↓; −2.29%; 0.05*	↔; 0.08%; 0.87	↓; 2.65%; 0.03*
Ergostik (*n* = 10)	↔; 0.18%; 0.49	↔; −0.45%; 0.07	↓; −1.51%; < 0.001*	↓; −1.02%; < 0.001*
VO2Master (*n* = 3)	↔; 0.78%; 0.49	↔; 0.16%; 0.92	—	—
PNOĒ (*n* = 3)	↔; −15.2%; 0.19	↓; −14.9%; 0.01*	↓; −15.0%; < 0.01*	↓; −13.5%; < 0.01*

*Note:* This analysis was only performed when two or more systems were available for a specific model. ↓ and ↑ represent a significant decrease and increase in the measured V̇O_2_ and V̇CO_2_ with higher simulated V̇O_2_ and V̇CO_2_, respectively, while ↔ represents no change. Note that decreases or increases can indicate that the error decreases depending on the error at the lower simulated value. For example, the error for V̇CO_2_ for Ergostik decreases with higher volumes due to the negative slope and initial positive value of the error.

Abbreviations: RER = respiratory exchange ratio; V̇CO_2_ = carbon dioxide production; V̇E = minute ventilation; V̇O_2_ = oxygen uptake.

* Significant slope *p* < 0.05.

### 3.2 Relationship System Age and Maintenance With Error

After removal of outliers (*n* = 3 for V̇E, *n* = 2 for V̇O_2_, *n* = 1 for V̇CO_2_, and *n* = 0 for RER), no significant correlations were observed between system age and the overall absolute percentage errors for any outcome, either when analyzed across all systems, or when each system was analyzed individually (Supplemental Table S1). These results did not meaningfully differ when outliers were included (maximum difference in correlation coefficient of 0.05). Note that the analysis per brand was only performed when at least five units were available to ensure somewhat robust correlations. The overall absolute percentage error did not significantly differ between systems receiving yearly maintenance (*n* = 44), and those not receiving yearly maintenance (*n* = 9) (−1.39%, *p* = 0.09).

### 3.3 Case‐Studies

**FIGURE 5:**
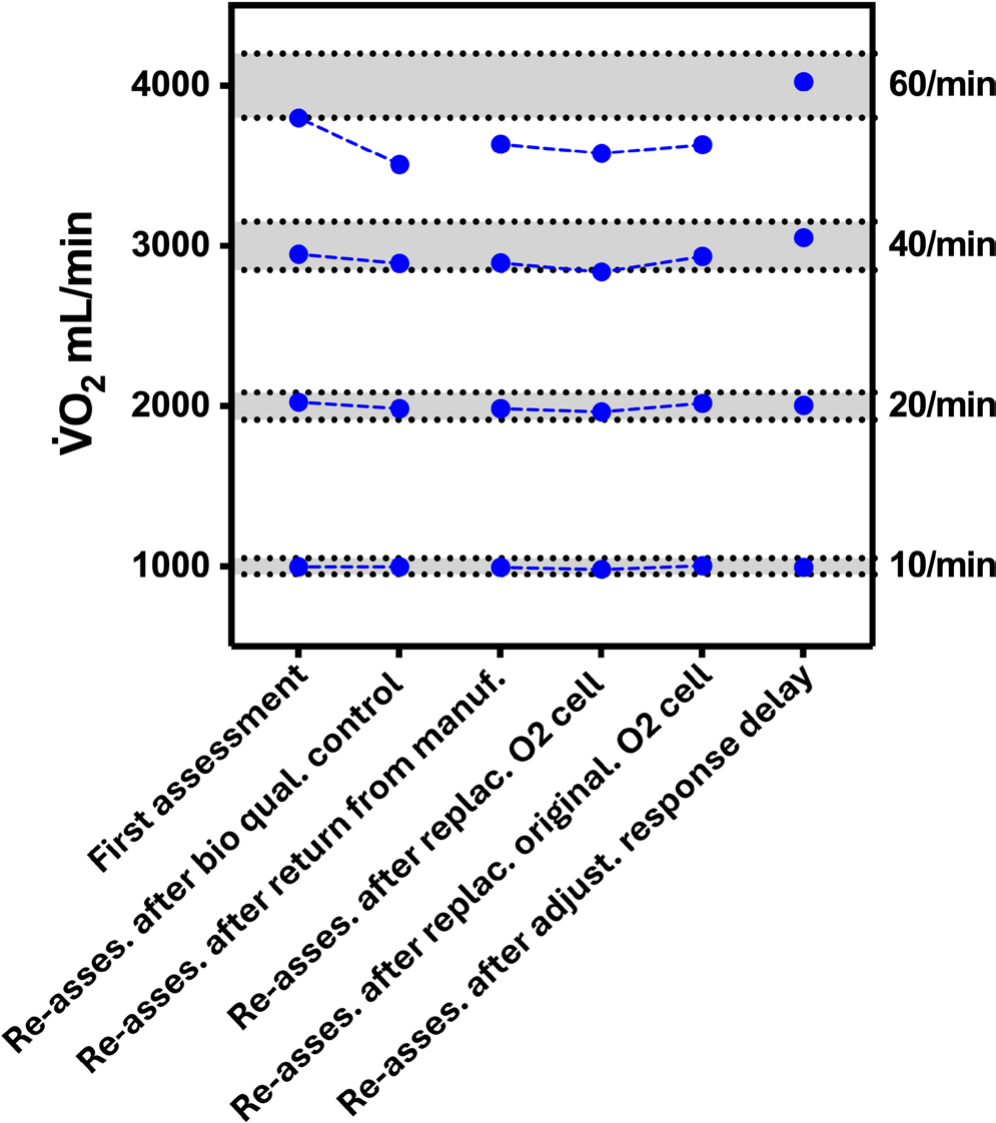
Case study showing the change in absolute percentage error across repeated simulations. Briefly, the first test shows V̇O_2_ values recorded during the CPX mode of the simulation for one system during the first assessment as part of this study. All values are within the acceptable range (50, 100, 150, 200 mL) as indicated by the shaded gray area. Four months later, biological quality control showed deviating V̇O_2_ values. Therefore, the metabolic simulation test was repeated to verify the observations during biological quality control. This confirmed that the V̇O_2_ at the highest breathing frequency was outside the acceptable range. Based on this assessment, the system was returned to the distributor/manufacturer. The distributor/manufacturer, however, found no reasons for inaccuracies and returned the system without adjustments. Reassessment resulted in another failure of V̇O_2_ accuracy, and the system was therefore once more returned to the manufacturer for repair. The manufacturer then replaced the O_2_ sensor, but the system still failed the simulation test. Similarly, the test failed when the old O_2_ sensor was again replaced. The system was therefore once again returned to the manufacturer, who adjusted the response time of the O_2_ sensor, resulting in acceptable accuracy. This case highlights the importance of quality control in detecting errors, even after replacement or repair procedures. V̇O_2_ = oxygen uptake.

Figure 5 shows the change in V̇O_2_ values for case 1, where repeated simulation assessments were performed. Briefly, repeated simulation assessments revealed a deficit in the O_2_ cell's response time, leading to errors in the recorded V̇O_2_ values. After correcting the response time, the V̇O_2_ values returned to the acceptable range.

**FIGURE 6:**
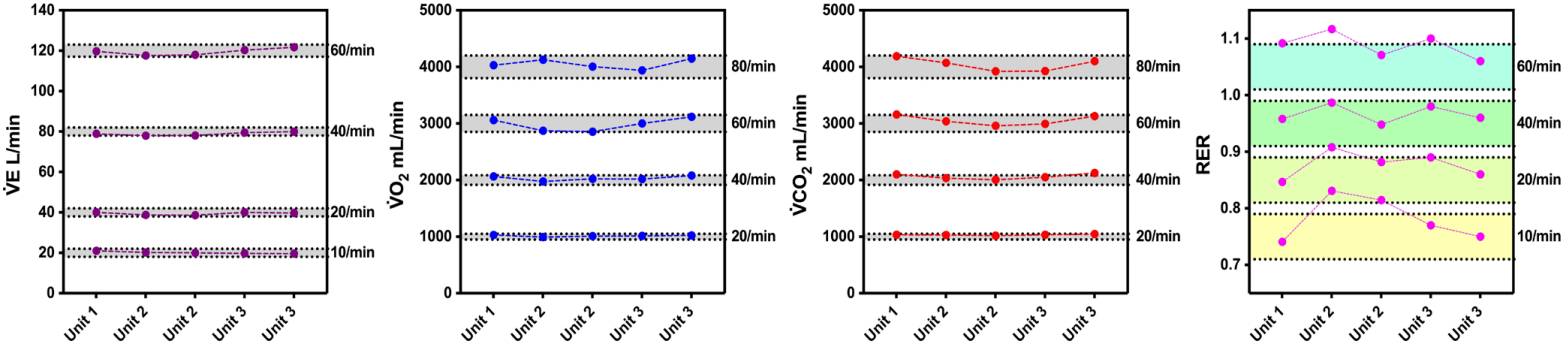
Variability in the accuracy among five different CPET units (from three manufacturers) within the same hospital for V̇E, V̇O_2_, V̇CO_2_, and RER. CPET = cardiopulmonary exercise testing, RER = respiratory exchange ratio, V̇CO_2_ = carbon dioxide production, V̇E = minute ventilation, V̇O_2_ = oxygen uptake.

Figure 6 further illustrates the variability in gas exchange values among five different CPET systems (from three manufacturers) within the same hospital. This comparison does not reflect inter‐unit variability among identical models, but rather differences between different brands of CPET systems at the same user location. Overall, these findings suggest that it is important to use the same unit for repeated testing to reduce trial‐to‐trial variability.

## 4 Discussion

This study aimed to (a) assess the in‐field validity of multiple commercially available CPET systems when operated by end‐users following typical calibration procedures, (b) evaluate inter‐unit variability of multiple identical CPET systems, and (c) examine the relationship between unit age, maintenance practices, and measurement accuracy.

The most important findings are that (a) there is a wide range in the accuracy of CPET systems used in the field, (b) there can be substantial inter‐unit variability of multiple identical CPET systems, and (c) there was no relationship between system age and overall accuracy. Absolute percentage errors for V̇O_2_, for instance, ranged from 3.29% to 10.6%, V̇CO_2_, from 2.86% to 13.3%, and RER from 1.90% to 10.0%. Similarly, models from the same manufacturer also ranged substantially in the measured values, with a range of 1.98%–12.7% for V̇O_2_, 1.49%–8.10% for V̇CO_2_, and 1.93%–4.24% for RER.

### 4.1 Overall Accuracy

The mean ± SD overall absolute percentage errors for V̇O_2_ and V̇CO_2_ were 4.44% ± 3.05% and 4.34% ± 3.18%, respectively (Table 2). When considering V̇O_2_, 40% of the systems assessed had a mean absolute percentage error < 3%, 72% < 5%, and 93% < 10% (Table 3). For V̇CO_2_, 42% of the systems assessed had a mean absolute percentage error < 3%, 70% < 5%, and 89% < 10%. This shows that, overall, most in‐field systems do not meet the threshold for good (< 3%) or acceptable (< 5%) accuracy, with some even showing very poor accuracy (> 10% error). Importantly, systems that on average show an absolute percentage error < 3%, may not necessarily show this during each simulated volume. Indeed, it was observed that the error for some systems systematically changed with changes in the simulated volumes and breathing frequencies (Figure 4). For example, the ~2.3% decrease in V̇O_2_ relative percentage error per step for the MetaMax translates into an underestimation of ~23 mL per 1000 mL increase in V̇O_2_. Although this magnitude is relatively small, it may be relevant in certain user application settings.

The absolute percentage errors were generally smaller in magnitude when computed over systems used in hospitals and rehabilitation centers compared to the average over all systems (Table 2). Similarly, more of the systems used in hospitals and rehabilitation centers met the < 3% or < 5% threshold (Table 3). This could reflect more rigid quality control procedures in hospitals and rehabilitation centers, but may also reflect the use of more accurate systems overall in these settings. Our findings primarily support the latter interpretation, as many hospitals and rehabilitation centers typically used the Ergostik and Vyntus CPX systems (Table 1), which previously also have been shown to exhibit lower error compared to for example systems more popular among trainers and coaches (VO2Master and PNOĒ; Table 2; although VO2Master was in the present study exclusively used by universities) [18]. It is interesting to note that the present findings suggest some wearable systems (e.g., VO2Master and PNOĒ) exhibit relatively higher overall errors than stationary systems, possibly due to space and weight constraints required for portability. However, this pattern was not consistent across all wearable devices, with for example MetaMax 3B showing largely similar accuracy to the stationary systems. Nevertheless, the observation that also within hospitals and rehabilitation centers only 55% and 38% of systems met the < 3% accuracy threshold for V̇O_2_ and V̇CO_2_, respectively—despite meeting the systems calibration checks—urges for more rigorous quality control procedures because the outcomes may be used for important clinical decisions [1, 2, 3, 4]. This finding is reinforced by previous findings from Xu and colleagues [34] who performed daily metabolic simulation assessment on eight different CPET systems from six manufacturers over the course of 5 years. Despite meeting the volume and gas concentration calibration checks by the manufacturer's software, systems still failed between 20% and 90% of the metabolic simulator validation assessments. Overall, these findings indicate a need for frequent quality control, not only by verifying if the system meets the manufacturer‐defined gas and volume errors in isolation, but also by assessing whether the measured combined respiratory outcomes (i.e., V̇O_2_ and V̇CO_2_) are accurate.

The accuracy differed between system brands and models, with Ergostik and Vyntus CPX generally showing the highest accuracy for the assessed outcomes, although the difference with Quark, MetaMax 3B, and MetaLyzer 3B was typically < 1% (Table 2 and Figure 2). It should be noted, however, that for some systems only a small number of devices were available (Table 1), thus providing a less precise indication of the overall accuracy. The observation of differences in accuracy between brands aligns with a recent study showing substantial differences between different CPET system brands when averaged across ~6000 individuals [42]. All but three systems exhibited an overall absolute percentage error for V̇E of < 3%, with two systems being close to 3% (Table 2). This suggests that errors may primarily arise from the measured gas concentrations or sensor response time rather than the measured volumes. Further, almost all systems showed a larger error in V̇CO_2_ than in V̇O_2_. In contrast to these findings, it has previously been argued that V̇O_2_ should have a higher error compared to V̇CO_2_ due to the Haldane transformation being required for calculating inspired volumes for V̇O_2_, but not for V̇CO_2_ [20]. Because the O_2_ sensor in most CPET systems degrades with use (e.g., oxidation of the anode metal and changes in electrolyte composition with exposure to oxygen), whereas the CO_2_ sensor does not, the higher V̇CO_2_ error unlikely reflects a faster degradation of the CO_2_ sensor compared with the O_2_ sensor. A more likely explanation for the higher error in V̇CO_2_ relates to the increase in environmental CO_2_ concentrations (and thus FiCO_2_) during the simulated exercise (due to the clinical/research staff and simulation itself) in combination with the device software algorithms. Specifically, during indoor lab test conditions, ambient CO_2_ rises (e.g., up to almost 0.2% in extreme cases [18]), while FiO_2_ decreases slightly. Some manufacturers however assume a fixed default ambient FiCO_2_ and FiO_2_ value (FiO_2_ ≈ 20.93%, FiCO_2_ ≈ 0.03%) and this may lead to an overestimation of the exhaled V̇CO_2_ when the inspired V̇CO_2_ value is already higher. The reason why V̇CO_2_ is more sensitive to this effect than V̇O_2_ is because ambient CO_2_ (FiCO_2_) is very low (~0.03%), and even small absolute changes (e.g., 0.05% to 0.15%) result in large relative errors (> 200%), whereas oxygen, with a much higher ambient fraction (FiO_2_ ≈ 20.9%), is less affected by similar small variations.

### 4.2 Inter‐Unit Technological Variability

Inter‐unit variability is a measure of how reproducible the results are when two or more identical units of a model are tested under identical situations [21]. To quantify inter‐unit variability, we computed the coefficient of variation (CV) from the inter‐unit standard deviation. Inter‐unit variability ranged from values similar to previous reports, to considerably higher values (CV 1.98%–12.7%, 1.49%–8.10%, and 1.41%–8.65% for V̇O_2_, V̇CO_2_ and V̇E, respectively; Table 4) compared with previous studies were technological variability (CV) between identical models was typically < 1.5% [19, 21, 24, 33]. However, all previous studies assessing inter‐unit differences used just two units of each model. Additionally, in at least two studies [19, 21], the units assessed were new and had been obtained very recently from the manufacturer. Over time, components such as oxygen sensors may degrade, thereby increasing inter‐unit differences, and possibly explaining the larger CVs in the present study. Specifically, repeated usage, sensor aging, and component drift can all contribute to a decline in accuracy over time. Investigating the rate and predictors of accuracy degradation could inform maintenance schedules and replacement timelines, thereby leading to more robust clinical and research environments. Our analysis showed, however, no consistent effect of age on the overall absolute percentage error (Table S1). Similarly, the accuracy did not differ significantly between systems receiving yearly maintenance versus those not receiving yearly maintenance. The lack of consistent significant relationships is likely explained by the interaction among multiple components contributing to accuracy. For example, as highlighted in the first case report, even new sensors may exhibit error independent of the sensor age (Figure 5 and Section 4.3). Furthermore, the age of a unit does not necessarily reflect the age of its individual sensors due to replacement and may therefore not correlate well with accuracy. Similarly, unit age does not capture usage frequency, which can affect sensor degradation. These findings therefore also suggest that the age of a system cannot be used to infer its accuracy, nor does it seem to consistently explain the variability in accuracy between systems.

In addition to sensor degradation, the variability between units can also result from differences in calibration, data processing, and hardware manufacturing tolerances. Specifically, a lack of calibration standardization across facilities may lead to inconsistencies, even among devices of the same manufacturer and model. For example, if users do not complete full strokes with the 3 L syringe during volume calibration, this may lead to a systematic overestimation of V̇E. Similarly, performing the syringe stroke too quickly or with too much impact at the end of a push/pull may exceed the tolerance range of the flow sensor, while a very slow stroke may not generate a sufficient signal for the sensor to detect flow; both of these conditions may lead to errors in the volume calculations, thus is the resulting measurement of all volume related parameters of CPET. Despite the possibility for errors in this calibration process, we do not believe this to be the primary reason for the observed inaccuracies, as questionnaires revealed most users used a relatively similar calibration procedure consisting of a warm‐up time of at least 30 min, as recommended by the manufacturer, followed by a gas and volume calibration in line with the manufacturer's guidelines. This included fully emptying the 3 L syringe, and the use of a stroke speed guided by the manufacturer's software, which usually includes criteria of acceptance within the calibration application software. Further, even systems that have an automated gas and volume calibration procedure (i.e., Vyntus CPX) reported some variability in accuracy between devices, although the magnitude of variability was smaller compared to systems that required manual calibration. Finally, our analysis showed that V̇E typically exhibited a much smaller inter‐unit variability than V̇O_2_ and V̇CO_2_ (Table 4). This suggests that variability in measured gas concentrations may account for more of the inter‐unit variability than V̇E, also suggesting a small role of volume calibration errors. In contrast, Macfarlane, Wu [21] previously found inter‐unit differences between two ParvoMedics systems to be primarily due to differences in V̇E rather than FeO_2_ or FeCO_2_. Similarly, Babineau and co‐workers [50] concluded that differences in V̇E were primarily responsible for differences observed in V̇O_2max_ when assessing the same subjects in different labs (albeit also using different systems). These differences may be explained by differences in the methodological set‐up of these studies (system age, ambient gas concentrations, etc.).

With regard to the possibility for errors in the gas calibration procedure, each facility also used the manufacturer‐recommended calibration gas, thus reducing the possibility of errors compared to gas sensor calibration with different precision grades of gases. However, calibration gas cylinders must be replaced once they are depleted, and new connected cylinders may contain gases with slightly different certified concentrations. The manufacturer's application software allows users to update the calibration gas settings accordingly when a new cylinder is installed. However, it was observed that at least four hospital departments had discrepancies between the gas concentration entered in the software and the concentration specified on the cylinder's certificate, which can introduce errors. These inconsistencies are best classified as user–application errors. Particular attention should therefore be given to this issue, especially in departments where multiple staff members are responsible for instrument calibration. Another example of user error is a facility where the module to measure ambient conditions was placed near a heat‐producing source, thereby also influencing CPET outcomes. Overall, these observations suggests that calibration may have some role, but also that factors other than calibration likely contributed to the differences in accuracy and inter‐unit variability.

Some facilities exhibited multiple CPET systems, with some being of the same brand and model, and others being of a different brand and model. When comparing the inter‐unit variability between three similar model units (Ergostik) from the same facility, an inter‐unit CV of 3.09% and 1.67% was found for V̇O_2_ and V̇CO_2_, respectively. For three Vyntus CPX units from the same facility an inter‐unit CV of 1.61% and 1.34% was found for V̇O_2_ and V̇CO_2_, respectively. These CVs are slightly smaller than the overall inter‐unit CV for both systems, suggesting units from the same facility are more similar than units from different facilities, possibly due to similar maintenance practices and use, as well as ambient testing environment. For Vyntus CPX, the inter‐unit variability is similar to the test–retest technological variability of the same unit [22], suggesting interchangeable use may be possible (at least within the specific facility used as an example here). In contrast, the CV of 3.09% for V̇O_2_ in the Ergostik could introduce additional noise beyond test–retest variability and suggests re‐assessments are preferably performed on the same model to reduce variability. This supports previous findings by Winkert et al. [33] who reported that the COSMED K5 inter‐unit variability exceeded intra‐unit test–retest variability and concluded that interchangeable use of different units could introduce additional noise to measurements.

### 4.3 Case Reports

We briefly highlight two case reports in which repeated CPET assessments were performed. In one situation, a system was re‐assessed that had just returned from the manufacturer for regular maintenance, but the facilities staff noticed a lower V̇O_2_ based on biological quality control. The CPET simulation assessment indeed revealed a relatively large (~4.5% absolute percentage difference) deviation in V̇O_2_, with the error also increasing proportionally with increases in simulated V̇O_2_ (Figure 5). Additional tests with a new O_2_ cell showed no improvement in the error. Because V̇E was relatively accurate, and because the error increased with higher breathing frequencies, we suspected the error to be related to an inaccurate O_2_ cell response time (e.g., based on findings reported by [51]). Indeed, after introducing a correction for the response time, the V̇O_2_ accuracy was within the acceptable range. Overall, this case study underscores the need for quality control to detect errors in CPET outcomes and to better identify possible causes of errors.

A second example also highlights the importance of quality control in understanding inter‐unit variability. Specifically, Figure 6 reports the simulation assessments from one hospital institution, in which three departments (pulmonary, sports medicine/cardiology, and outpatient clinic) used five CPET systems from three different manufacturers. Although primary indices like V̇E, V̇O_2_, and V̇CO_2_ for all individual systems appear to be within acceptable ranges, visual inspection shows that system‐to‐system differences increase at higher intensities and may be as high as 5% for V̇O_2_, V̇CO_2_. These differences are even more evident in RER, where some values are outside the acceptable range due to errors in V̇O_2_ and V̇CO_2_ being in opposite directions. Such differences could influence for example staff decisions on ending a maximum CPET procedure for a participant across different departments. These findings match with a recent study showing differences of up to 17% in V̇O_2_ at the first ventilatory threshold between different brand devices at the same facility, despite all devices having been checked by the manufacturers prior to the study, and meeting the manufacturer calibration checks [52]. This variability between systems underscores the importance of using the same brand and model unit for repeated human testing to reduce variability.

### 4.4 Limitations

This study has several strengths, but also some limitations. Strengths include the assessment of accuracy across a relatively large number of devices and the in‐field setting, with calibration and operation by staff who would also typically perform CPET assessments in the field. This latter aspect increases the relevance of the findings to clinical practice or multi‐center scientific studies. A first limitation is that a metabolic simulator does not fully match human exercise because it uses dry gases, a lower temperature of gases, and a sinusoidal breathing pattern [18, 53]. While verification during human exercise testing would have been beneficial to confirm the findings obtained with simulation, this was not feasible given the large number of systems assessed. Previous studies have, however, generally observed deviations in simulation experiments to match those in human exercise testing [18, 19, 22], thus increasing confidence in the findings with simulation testing for human exercise testing. In the present study, the deviation in biological quality control (Figure 5) was also seen during simulation, thus further reinforcing this notion. In this context, the present simulator is limited to a V̇O_2_ of 4 L·min^−1^, leaving the performance of the CPET systems at higher intensities, such as those encountered by highly trained individuals, unknown. A second limitation is that the metabolic simulator assumes a certain distribution of atmospheric gases (i.e., N_2_ of 79.05%, O_2_ 20.9%, and CO_2_ 0.05%) when adding N_2_ and CO_2_ to the inhaled gas mixture, and deviations from the assumed distribution can introduce errors in the simulated gas exchange data (see Supplemental file in [22] for more details). Similarly, variability in the atmospheric gases across different user settings can also introduce variability between repeated measures. However, we anticipate the errors and variability caused by this to be small (< 0.5%) because environmental conditions (pressure, temperature, relative humidity, CO_2_ concentration) were monitored and verified to be relatively similar and within the simulator specified operating range across the different facilities. Third, we did not have detailed information on aspects such as the number of tests performed with each system since purchasing, or the age of the O_2_ sensor, thus not allowing us to assess the relationship between these aspects and system accuracy. Fourth, all systems were used in breath‐by‐breath mode. Additional inter‐unit variability may be introduced when one device in used in breath‐by‐breath mode, and another in mixing chamber mode. Finally, our study included systems primarily located in the Netherlands and may not be generalized to different brands and models used in different countries. However, in line with the present study, a survey of 84 pediatric CPET centers in the US reported the Vyntus CPX system was most commonly used [54], with other recent findings also indicating frequent use of this system across different sites across the world [42] thus suggesting wider utility of our findings. Nevertheless, for some systems, we were able to include only a small number of units, thus reducing our ability to accurately assess inter‐unit variability.

## 5 Conclusion

The accuracy of metabolic carts for assessing respiratory gas variables differed substantially between different CPET brands, but also between identical systems from the same brand, despite meeting manufacturer calibration checks. Regular quality control should be used to verify system accuracy. Care should be taken with interchangeable use of different systems as this may introduce additional noise in measurements.

## 6 Perspective

Accurate assessment of respiratory gases is critical because they are used to make clinical decisions [1, 2, 3], guide exercise prescription [5, 6], and support a wide range of research applications [7, 8, 9, 10, 11, 12, 13, 14, 15, 16]. Minor errors can lead to misclassification of a patient's cardiorespiratory fitness status or incorrect estimation of exercise thresholds, potentially impacting diagnoses or therapeutic decisions. Given that many manufacturers claim a 3% accuracy threshold, it is notable that few systems actually achieve this in day‐to‐day practice, which raises broader questions about regulatory standards, manufacturer calibration/validation protocols, and the real‐world utility of published device specifications. Most inaccuracies seemed related to a combination of factors on the actual performance of a device (e.g., sensorics), although some user application errors were also identified, suggesting a holistic approach should be used to identify possible errors. For example, we observed some errors to stem from user‐errors (incorrect specification of calibration gas specifications in the system), while other errors present technical errors (e.g., O_2_ cell response time). Regardless of the cause, regular quality control may aid in detecting these errors, as documented in this study. This is similar to previous observations of a biological quality control procedure implemented in 15 different centers, with eight centers initially failing the biological quality control procedure, requiring system maintenance [55].

Inter‐unit variability differed considerably, despite all systems meeting the manufacturer's gas concentration and volume calibration assessments. These findings suggest care with interchangeable use of different systems, even from the same brand and model, as this may introduce additional variability that could lead to incorrect conclusions in a personalized medicine approach or reduce statistical power in a research setting. Indeed, some previous findings suggest relatively large inter‐unit variability [56]. For example, Yule et al. [56] reported V̇O_2max_ varied by 4%–14% between three labs using a similar model (Sensormedics 2900) CPET system. Similarly, the HERITAGE Family study assessed the reproducibility of physiological variables during two submaximal and one maximal test in the same eight participants across four laboratories and reported coefficients of variation (CVs) for V̇O_2_ of 3.5% and 4.1% during submaximal [57] and maximal [58] testing, respectively. While it has been argued that the majority of the variability in these studies reflects biological variability due to repeated human testing [21, 22], our findings suggest that technological inter‐unit variability may also contribute meaningfully to the actual test variability in some units (Table 4). This finding may also partly explain the large variability in V̇O_2max_ observed across different sites world‐wide [42]. Accordingly, the present findings highlight the need for future efforts to improve inter‐laboratory comparability, including the development and implementation of standardized quality‐control frameworks, cross‐center certification procedures, and harmonized validation protocols for both multicenter research and clinical centers.

An important consideration when interpreting our findings relates to the calibration procedure in the present sample of users. Specifically, all users performed a calibration prior to the simulation assessment. However, a survey among 84 pediatric CPET centers in the United States showed that while the majority of centers perform calibration prior to each test (~70% gas calibration, ~50% flow calibration), some users perform calibration only once daily or a small minority even weekly [54]. The error observed in the present study may therefore increase further due to sensor drift when calibration is done less regularly. When multiple tests follow one another, we also strongly recommend repeating volume and gas calibration before each test to optimize system accuracy. Further, PNOE was assessed after calibrating with calibration gas. It has previously been shown that this procedure increases accuracy compared with the air calibration recommended by the manufacturer [18], and the error for this system therefore increases when using this practice.

## Funding

Funding was received from the Dutch Federation for Sports Medical Facilities (Federatie voor Sport Medische Instellingen). Relitech BV provided a metabolic simulator and the necessary gases for the experiments described in this manuscript.

## Conflicts of Interest

The authors declare no conflicts of interest.

## Supporting information


**Table S1:** Relationship system age and overall absolute percentage error.

## Data Availability

All meta‐data is available from the Open Science Framework at: https://doi.org/10.17605/OSF.IO/HF3UA.
