# Supplementary material for: In‐Field Validity and Inter‐Unit Variability of Metabolic Carts During Simulated Exercise
Source: Scand J Med Sci Sports. 2026 May 6;36:e70297. doi: 10.1111/sms.70297 (PMC13150039; doi:10.1111/sms.70297)
Supplement: Supplementary file 1 — Table S1: Relationship system age and overall absolute percentage error. [file SMS-36-e70297-s001.docx]

**Supplemental file**

**Table S1. Relationship system age and overall absolute percentage error**

| **System/outcome** |  |
| --- | --- |
| **All systems** | **p-value linear mixed model** |
| V̇E | p 0.95 |
| V̇O_2_ | p 0.37 |
| V̇CO_2_ | p 0.86 |
| RER | p 0.55 |
|  |  |
| **Vyntus CPX** | **Correlation coefficient; p-value linear regression** |
| V̇E | -0.40; p 0.14 |
| V̇O_2_ | 0.06; p 0.84 |
| V̇CO_2_ | 0.33; p 0.25 |
| RER | -0.001; p 0.99 |
|  |  |
| **Ergostik** |  |
| V̇E | -0.46; p 0.21 |
| V̇O_2_ | -0.19; p 0.63 |
| V̇CO_2_ | -0.28; p 0.46 |
| RER | -0.36; p 0.34 |
|  |  |
| **Quark CPET** |  |
| V̇E | -0.41; p 0.31 |
| V̇O_2_ | 0.28; p 0.50 |
| V̇CO_2_ | 0.43; p 0.28 |
| RER | 0.58; p 0.13 |
|  |  |
| **MetaLyzer 3B** |  |
| V̇E | 0.20; p 0.49 |
| V̇O_2_ | 0.43; p 0.12 |
| V̇CO_2_ | -0.11; p 0.72 |
| RER | 0.33; p 0.25 |
|  |  |
| **MetaMax 3B** |  |
| V̇E | 0.24; p 0.76 |
| V̇O_2_ | 0.44; p 0.56 |
| V̇CO_2_ | 0.22; p 0.78 |
| RER | 0.64; p 0.36 |
